# Tumor sequencing is useful to refine the analysis of germline variants in unexplained high-risk breast cancer families

**DOI:** 10.1186/s13058-020-01273-y

**Published:** 2020-04-15

**Authors:** Cédric Van Marcke, Raphaël Helaers, Anne De Leener, Ahmad Merhi, Céline A. Schoonjans, Jérôme Ambroise, Christine Galant, Paul Delrée, Françoise Rothé, Isabelle Bar, Elsa Khoury, Pascal Brouillard, Jean-Luc Canon, Peter Vuylsteke, Jean-Pascal Machiels, Martine Berlière, Nisha Limaye, Miikka Vikkula, François P. Duhoux

**Affiliations:** 1 Department of Medical Oncology, Institut Roi Albert II, Cliniques universitaires Saint-Luc and Institut de Recherche Expérimentale et Clinique, UCLouvain, Brussels, Belgium; 2 Human Molecular Genetics, de Duve Institute, UCLouvain, Brussels, Belgium; 3 Center for Human Genetics, Cliniques universitaires Saint-Luc, Brussels, Belgium; 4 Breast Clinic, Institut Roi Albert II, Cliniques universitaires Saint-Luc, Avenue Hippocrate 10, 1200 Brussels, Belgium; 5 Laboratory of Translational Oncology and IPG BioBank, Institute of Pathology and Genetics, Gosselies, Belgium; 6 Center for Applied Molecular Technologies, Institut de Recherche Expérimentale et Clinique, UCLouvain, Brussels, Belgium; 7 Department of Pathology, Cliniques universitaires Saint-Luc, Brussels, Belgium; 8 Department of Pathology, Institute of Pathology and Genetics, Gosselies, Belgium; 9 Breast Cancer Translational Research Laboratory, Institut Jules Bordet, Université Libre de Bruxelles, Brussels, Belgium; 10 Genetics of Autoimmune Diseases and Cancer, de Duve Institute, UCLouvain, Brussels, Belgium; 11 Department of Oncology-Hematology, Grand Hôpital de Charleroi, Charleroi, Belgium; 12 Department of Medical Oncology, UCLouvain, CHU UCL Namur, site Sainte-Elisabeth, Namur, Belgium

**Keywords:** Breast cancer, Predisposition, Germline, Second hit, Variant of unknown significance, Mutational signatures

## Abstract

**Background:**

Multigene panels are routinely used to assess for predisposing germline mutations in families at high breast cancer risk. The number of variants of unknown significance thereby identified increases with the number of sequenced genes. We aimed to determine whether tumor sequencing can help refine the analysis of germline variants based on second somatic genetic events in the same gene.

**Methods:**

Whole-exome sequencing (WES) was performed on whole blood DNA from 70 unrelated breast cancer patients referred for genetic testing and without a *BRCA1*, *BRCA2*, *TP53*, or *CHEK2* mutation. Rare variants were retained in a list of 735 genes. WES was performed on matched tumor DNA to identify somatic second hits (copy number alterations (CNAs) or mutations) in the same genes. Distinct methods (among which immunohistochemistry, mutational signatures, homologous recombination deficiency, and tumor mutation burden analyses) were used to further study the role of the variants in tumor development, as appropriate.

**Results:**

Sixty-eight patients (97%) carried at least one germline variant (4.7 ± 2.0 variants per patient). Of the 329 variants, 55 (17%) presented a second hit in paired tumor tissue. Of these, 53 were CNAs, resulting in tumor enrichment (28 variants) or depletion (25 variants) of the germline variant. Eleven patients received variant disclosure, with clinical measures for five of them. Seven variants in breast cancer-predisposing genes were considered not implicated in oncogenesis. One patient presented significant tumor enrichment of a germline variant in the oncogene *ERBB2*, in vitro expression of which caused downstream signaling pathway activation.

**Conclusion:**

Tumor sequencing is a powerful approach to refine variant interpretation in cancer-predisposing genes in high-risk breast cancer patients. In this series, the strategy provided clinically relevant information for 11 out of 70 patients (16%), adapted to the considered gene and the familial clinical phenotype.

## Introduction

Hereditary forms of cancer have been described for decades. Evidence-based guidelines for screening are now applied for suspected hereditary breast and ovarian cancer (HBOC) syndrome, Lynch syndrome, and other conditions [1, 2]. Screening multiple genes simultaneously by massively parallel sequencing is cost-effective and has replaced single-gene sequencing in hereditary breast cancer (HBC). It can reveal mutations in clinically validated genes in up to 5% of cases without *BRCA1* or *BRCA2* mutations [3]. Its use will probably expand, as recent publications question the validity of established screening criteria given the high number of germline mutations identified in cancer types unrelated to the initial syndrome or in patients lacking family history [4, 5]. However, multigene panel testing has a major drawback: the likelihood of identifying a variant of unknown significance (VUS) far exceeds that of discovering a pathogenic mutation, especially as the number of genes tested increases [6]. Indeed, several converging arguments are required to define pathogenicity of a variant [7, 8].

Taking VUS into consideration is a daily clinical challenge. It has a major impact on the preventive screening or treatment strategy; therefore, misinterpretation of a VUS can be physically or psychologically harmful [9]. Functional testing helps reclassify VUS and is trending in translational studies [10], but feasibility on a clinical scale is sparse and not yet implemented [11]. Large international consortia like ENIGMA aim to reclassify variants by gathering genotypic and phenotypic data from various sources, recognizing that the rarity of the variants is the main issue [12].

Current variant classification guidelines do not include analysis of matched tumor samples. Yet, the two-hit theory for inherited cancer predisposition conferred by heterozygous, germline mutations in tumor suppressor genes postulates that the normal allele is locally lost or outcompeted by the mutant allele, due to a second, somatic variation in the same gene. These may be copy number alterations (CNAs), pathogenic point mutations, small insertions/deletions (INDELs), or epigenetic modifications that reduce the expression or function of the normal allele, or increase that of the germline mutant [13]. We therefore hypothesized that matched tumor sequencing could serve as an argument to study the implication of germline variants in the development of cancer in HBC patients, based on the presence of somatic events in the same gene.

## Methods

### Patients and germline DNA samples

Patients were eligible for inclusion if they had a personal history of breast and/or ovarian cancer, met the criteria for clinical genetic counseling and testing based on the guidelines of the Belgian Society of Human Genetics and were negative for *BRCA1*, *BRCA2*, *TP53*, and *CHEK2* pathogenic mutations. Matching tumor material had to be available. All patients signed an informed consent approved by the Ethics Committee of the hospital. Demographic, familial, and clinical data were recorded to calculate the breast cancer (BC) lifetime residual risk and *BRCA* mutation carrier pre-test probability for each patient using the BOADICEA algorithm [14]. Ten milliliters of blood was drawn from each patient for DNA extraction using the Wizard genomic DNA purification kit (Promega).

### Germline whole-exome sequencing (WES)

Briefly, 1 μg of genomic DNA was processed. Genomic DNA was captured using Agilent in-solution enrichment methodology with their biotinylated oligonucleotide probes library (SureSelect V6 Exome, Agilent Technologies), followed by paired-end 150 bases massively parallel sequencing on Illumina HiSeq4000 to at least 60× average coverage. Sequence capture, enrichment, and elution were performed according to the manufacturer’s instructions and protocols. Image analysis and base calling were performed using Illumina Real-Time Analysis (2.7.7) with default parameters.

### Matched tumor WES

Five consecutive 10-μm sections were obtained from the most tumor-representative formalin-fixed and paraffin-embedded (FFPE) sample. A matched hematoxylin and eosin-stained section was used to macrodissect the tumor area. DNA was extracted using the QIAamp DNA FFPE Tissue Kit (Qiagen) and quantified using the Qubit dsDNA high-sensitivity Assay kit (Thermo Fisher Scientific). Tumor WES was performed by Integragen (Ivry, France) with similar capture kit and sequencer as the germline WES. Specifically, a minimum amount of 50 ng of DNA was needed to create the libraries, followed by paired-end 75 bases massively parallel sequencing to at least 120× average coverage.

### Bioinformatics processing of WES data

Both germline and tumor reads were aligned to the reference human genome sequence GRCh37 using Burrows-Wheeler Aligner 0.7.15 (Wellcome Trust Sanger Institute). Duplicate reads were marked and removed using Picard 1.107 (Broad Institute). Local realignment around indels and base quality score recalibration were performed using the Genome Analysis Toolkit 3.3 (Broad Institute). Germline single-nucleotide variants (SNV) and small indels were identified using GATK Haplotype Caller 3.3 whereas somatic SNV and small indels were identified using the Mutect2 algorithm based on GATK Haplotype Caller 3.7 (Broad Institute). Called variants were annotated, filtered, and visualized using Highlander (http://sites.uclouvain.be/highlander/), an in-house bioinformatics framework.

### Classification and selection of germline variants identified by WES

We used a list of 735 candidate genes, including 565 genes selected for germline mutation analysis in a previous landmark study of cancer predisposition [15], supplemented with genes implicated in DNA repair or related to BC by literature mining (Table S1). Variants were retained if passing quality criteria (Phred score for quality of mapping > 30, no more than two different haplotypes at the considered position, variant called outside the 3′ end of the supporting reads, absence of strand bias) had an allele frequency in the ExAC database of < 0.015, were considered pathogenic by at least two prediction softwares (among SIFT, CADD, Fathmm, LRT, DEOGEN2, Mutation Assessor, Mutation Taster, and Polyphen2), or affected splicing (estimated by 2 ensemble learning methods [16]).

Germline variants in well-established BC predisposing genes (*BRCA1*, *BRCA2*, *TP53*, *PALB2*, *ATM*, *CHEK2*, *CDH1*, *PTEN*, *STK11*) were manually classified according to ACMG guidelines [17]. Variants classified as VUS, pathogenic, or likely pathogenic were retained, as were the variants with conflicting interpretation in ClinVar [18]. Germline variants in the remaining genes (thus without known association with the phenotype) were retained if meeting the aforementioned sequencing filtering criteria.

### Assessment of tumor WES data for somatic second hits

We assessed each tumor for somatic variations (point mutations; INDELs; CNAs, i.e., amplifications and deletions; loss of heterozygosity (LOH); and copy-neutral loss of heterozygosity (CN-LOH)) in the genes containing a germline variant. The presence (both positive as negative) or absence of selection pressure of the germline variant in the tumor sample was assessed by the difference in allele balance between tumor and normal (DAB) analysis (chi-square test, *p* value threshold of 0.05 for significance) derived from the allelic depths in both samples. DAB of the considered genomic region was further considered by analysis of the behavior in the tumor of each germline heterozygous SNV on the given chromosome. The Benjamini-Hochberg procedure was used to correct for multiple testing, and *p* values were plotted as in Manhattan plots from genome-wide association studies. True DAB was retained only if the region surrounding the germline variant depicted significant *p* values for DAB. CNAs were assessed by the FACETS algorithm [19]. Regarding the locus of the germline variant, loss of heterozygosity (LOH) was defined as the loss of the normal allele in the tumor. Copy-neutral LOH (CN-LOH) was defined as a diploid status with DAB in the tumor. Homozygous deletion (HZ-DEL) was defined as the loss of both alleles. Amplification was defined as a copy number status ≥ 6, similar to the threshold used when considering clinically meaningful ERBB2 amplification [20]. The validity of the allele calls was cross-checked with the DAB analysis. Somatic mutations in a gene carrying a considered germline variant were called using Mutect2, as described above, and retained only if the germline variant did not display negative pressure selection in the tumor.

### Analysis of the patterns of somatic mutations

Global patterns of somatic variants were analyzed using complete WES data. We analyzed mutational signatures and quantified the contribution of the known COSMIC signatures (http://cancer.sanger.ac.uk/cosmic/signatures) to the observed somatic mutational processes using the R package MutationalPatterns [21]. Homologous recombination deficiency (HRD) was determined in each tumor sample. We processed the data derived from FACETS to calculate three different HRD scores (telomeric DAB, large-scale state transition, and genomic LOH) combined to a global mean HRD score, using the R scripts kindly made available by Nathanson and Pluta et al., described elsewhere [22]. We used MutSigCV to identify significantly mutated genes [23]. Tumor mutation burden (TMB) was defined as the ratio of the number of somatic variants detected (after the exclusion of germline variants) and the size of the capture kit (60 Mb).

### Visualization of the genomic results

All analyses downstream of the variant calling were performed in R (version 3.5.1, http://www.R-project.com). Data visualization was obtained with in-house developed scripts, with the Gviz and GenVisR packages [24, 25], or with ProteinPaint [26].

### Evaluation of splicing alterations

RNAs were extracted from lymphocytes with TriPure (Roche) and retro-transcribed using RevertAid H-Minus First Strand cDNA Synthesis Kit (Fermentas), with random hexamers. PCR amplification was done using specific primers, available upon request. Amplicons were cloned into pCRII-TOPO Vector (Invitrogen). Plasmids were purified with PureYieldTM Plasmid Miniprep System (Promega) and Sanger sequenced.

### In vitro kinase assay of the germline ERBB2 variant

MSCV-human Erbb2-IRES-GFP was a gift from Martine Roussel (Addgene plasmid # 91888; http://n2t.net/addgene:91888; RRID:Addgene_91,888) [27] and served as a template for mutagenesis (the considered variant and the positive control V777L described in Bose et al. [28]). Primers for the mutagenesis (available upon request) were designed using QuikChange Primer Design (Agilent). The entire coding sequence was verified using Sanger sequencing before and after the insertion in a lentiviral vector. HEK293T cells were grown in Dulbecco’s modified Eagle’s medium (DMEM) supplemented with 10% fetal bovine serum and 1% penicillin/streptomycin. As the overexpression of wild-type ERBB2 has an oncogenic effect which could prevent us from seeing the effect of the mutations, we artificially reduced the number of ERBB2 proteins expressed in each cell, by transfecting a mix (5% ERBB2 plasmid and 95% of empty lentiviral vector) of plasmids into the HEK293T cells using jetPEI® (Polyplus, France) according to the manufacturer’s instructions. Protein lysates were homogenized using a 21-gauge needle and resolved on precast polyacrylamide gels (Bio-Rad). Primary antibodies were purchased from Cell Signaling Technologies: ERBB2, phospho-ERBB2(Y1248), EGFR, phosphor-EGFR(Y1068), phospholipase C gamma (PLCγ), phospho-PLCγ(Y783), and alpha-actinin and used at recommended dilutions. Separate membranes were used for total and phospho-antibodies. Visualization was performed with an anti-rabbit secondary antibody (BioSource) at 1:10,000 dilution with a femto-sensitive ECL detection system (Pierce).

### Immunohistochemistry

Immunohistochemistry was performed on 4-μm paraffin sections. Heat-induced antigen retrieval was performed in a PT-link pre-treatment module (DAKO, Agilent Technologies). After endogenous peroxidase blocking, sections were incubated overnight at 4 °C with a PMS1 rabbit polyclonal primary antibody (1:100 dilution, 10859-1-AP, Proteintech). After three washes with TBS-Tween, sections were incubated with HRP-conjugated anti-rabbit polymer (Envision, DAKO) for 30 min at room temperature, and immunoreactivity was revealed using 3′3′-diaminobenzidine.

## Results

### Population

We could collect germline and tumor DNA for 70 unrelated BC patients. Patient characteristics are summarized in Table 1, depicting a population at high risk for HBC. Only one patient was male.

**Table 1:** Clinical characteristics of the study participants

	70 patients
**Age at diagnosis**, mean ± SD (range)	46 ± 11 years (26–79)
**Relatives with breast cancer**, mean ± SD (range)	4.1 ± 1.5 (1–9)
**Histology**	
- Ductal carcinoma	84%
- Medullary carcinoma or medullary-like	4%
- Other invasive carcinomas	8%
- Ductal in situ carcinoma	4%
**Grade**	
- Grade 1	13%
- Grade 2	55%
- Grade 3	27%
- Missing	5%
**Size**, median (range)	17 mm (2–115)
**Ki67**, median (range)	20% (5–80)
**Estrogen receptor +**	75%
**Molecular classification**	
- Luminal (A - B - ERBB2+)	52% (15% - 34% - 16%)
- ER− ERBB2+	3%
- Triple-negative	13%
- ERBB2 status missing	19%
**Positive lymph nodes**	34%
**2nd breast cancer** (of which contralateral)	34% (81%)
**Breast cancer lifetime residual risk**, mean ± SD (range)	15 ± 7% (2–30)*—*26 NA
**BRCA mutation carrier pre-test probability**, median (range)	13% (1–91)

Breast cancer lifetime residual risk and BRCA mutation carrier pre-test probability as determined by BOADICEA [14]. Breast cancer lifetime residual risk cannot be assessed for patients having already presented bilateral breast cancer and ovarian cancer or > 80 years old

*SD* standard deviation

### Germline variants

We identified 329 rare variants in 139 genes across the 70 patients (mean ± SD, 4.7 ± 2.0; range, 0–12): 293 (89%) missense SNVs or in-frame INDELs, 12 (4%) nonsense SNVs or frameshift INDELs, and 24 (7%) splice region variants (Table S2). Missense variants were predicted pathogenic by 4.5 ± 1.8 algorithms (mean ± SD). In two patients (including the sole male), no variant satisfied our filtering criteria. There were 18 variants in a well-established BC-predisposing gene. Of these, only one was a known pathogenic variant (*PALB2* NM_024675 c.509_510delGA).

### Difference in allele balance between tumor and normal

We detected a possible DAB of the variant (i.e., significantly different ratio of variant to reference allele, in tumor compared to blood) for 57 (17%) variants. This was confirmed by chromosome-wide SNV allele ratio analysis for 36 variants (11%), in 22 patients (31%) (Figure S1), including the *PALB2* pathogenic mutation.

### Analysis of somatic second hits in tumor WES

Genome-wide aneuploidy (cumulative size of amplifications, LOH, and deletions) in the tumors ranged from 0.1 to 56% (median 13.6%). Per tumor, aneuploidy covered 3–114 (median 32.5) of the 735 genes.

**Fig. 1:**
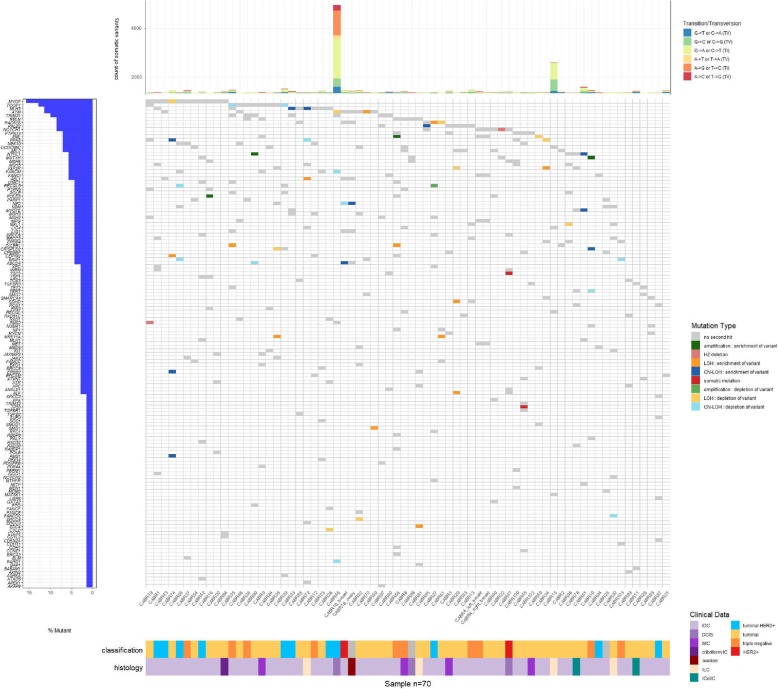
Waterfall plot depicting the presence (and type) or absence of a somatic second hit for the germline variants in the matched tumor samples

Somatic variation at the genomic coordinates of the 329 germline variants is depicted in Fig. 1 (lower panel). Fifty-five germline-mutated genes (16% of the retained variants) from 32 patients presented a somatic CNA or second mutation in the matched tumor. Of the 55-s hits, two were somatic second mutations: *TSC2* NM_000548 c.774G>C in the tumor of CABR47, but presumably a passenger mutation (8% allele frequency (AF)); *TP53* NM_001126112 c.365_366delTG in the tumor of CABR45, likely the driver mutation (20% AF). Of the 53 somatic CNAs, 28 would result in enrichment and 25 in depletion of the germline variant in the tumor. Genome-wide aneuploidy size did not differ between the samples with or without a somatic second hit (Mann-Whitney *U* test, *p* = 0.80).

### Refining the analysis of germline variants on the basis of global somatic mutation patterns

**Fig. 2:**
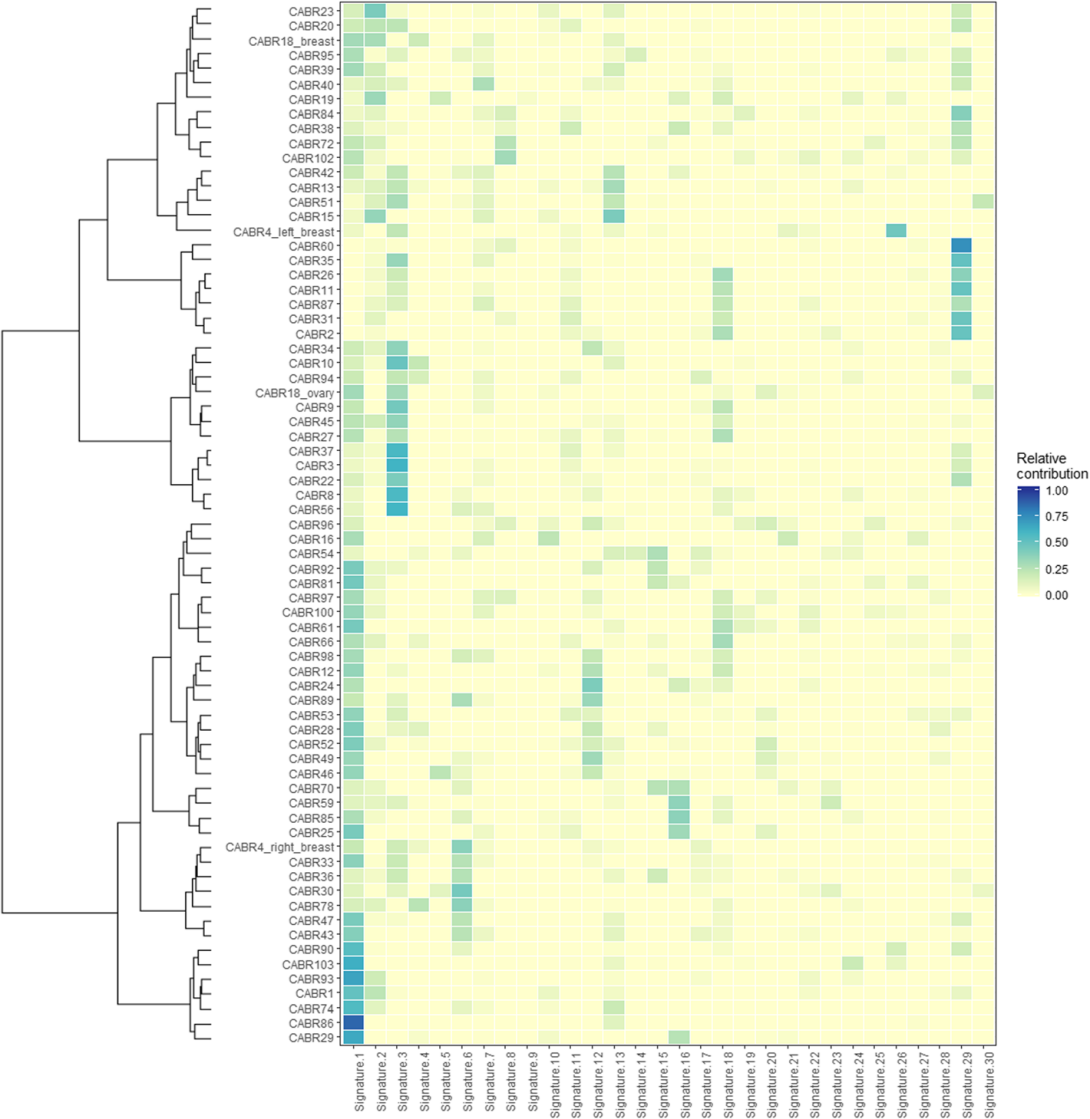
Mutational signatures operative in the tumor samples, depicted as the optimal relative contribution of COSMIC signatures to reconstruct the mutational profiles of the samples

We next broadened our analysis to the global patterns of somatic variation observed in tumor data and assessed for whether these patterns could also be exploited to prioritize additional variants. CABR95, carrying the germline pathogenic *PALB2* mutation enriched in the tumor by somatic LOH, had a high mean homologous recombination deficiency (HRD) score, in keeping with the expected effects of loss-of-function of this gene (Figure S2A). CABR46 carried an interesting candidate germline variant in *PMS2* (NM_000535 c.1937G>T): rare (minor allele frequency (MAF) 0.012% in the European non-Finnish population, with no homozygotes in the GnomAD database) and predicted damaging by 6 algorithms. No somatic second hit in *PMS2* was detected in the matched tumor sample. Nevertheless, this tumor was hypermutated (5444 somatic variants, tumoral mutation burden (TMB) of 90 variants/Mb) (Fig. 1, upper panel) and had the highest number of indels in the series (Figure S3). C>T transitions predominated (44%), and signatures 6 and 20 (related to mismatch repair deficiency) accounted for 15% of the tumor mutational profile in this sample mainly characterized by signatures 1 and 5 related to aging and deamination, respectively (Fig. 2). Although no second hit was found, these data orient towards the probable existence of mismatch repair deficiency, supporting a causal role for the *PMS2* variant and the presence of a second hit of a different type (e.g., an epigenetic event). The detection of microsatellite instability through the exome data, using MSIsensor [29], was also not definitely conclusive; no sample reached a score of 10 considered in one study to define microsatellite instability [30]. Nevertheless, CABR46 had an outlier score as compared to the other samples (1.3 vs 0.4, Figure S4), as did an in-house positive control case of breast cancer with proven microsatellite instability (3.2, Schröder et al., unpublished). The exhaustion of tumor material unfortunately prevented us from quantifying microsatellite instability using PCR amplification of validated microsatellites loci. In contrast, CABR19, who had bilateral BC before 38 years of age and carried an even stronger germline candidate variant on *PMS2* (c.883C>T; one occurrence in the European non-Finnish population in GnomAD, predicted damaging by all algorithms) had low TMB (1.4/Mb) and somatic indel count [4], a very low MSIsensor score (0.01), and did not display signatures 6 and 20. This supports the absence of a somatic second hit inactivating PMS2.

CABR51 had the third most highly mutated tumor (395 somatic variants of which 47% were C>T transitions, TMB of 6.6 variants/Mb) with the second-highest number of indels (Figure S3). This could be related to the germline variant on *NTHL1* (NM_002528 c.527 T>C; MAF 0.21% in GnomAD including only one homozygote, and predicted damaging by all algorithms), enriched in the tumor by CN-LOH. Signature 30 was the main contributor to this somatic mutational profile, a feature unique to this tumor sample. In contrast, CABR90 also had a germline *NTHL1* variant (c.298 T>C; MAF 0.12% in GnomAD, predicted damaging by 7 algorithms), but with a balanced amplification of the region in the tumor. NTHL1 is expected to act as a tumor suppressor gene, arguing against amplification as a bona fide second hit. This was underscored by the low TMB (1 variant/Mb), low indel count (1 indel), and the absence of signature 30 in this tumor.

### Concordance with known breast cancer genomic data

Despite the suboptimal conservation of the FFPE tumor samples, the validity of the WES data was strongly supported by expected observations. Mutational signatures related to aging (signature 1), activity of the APOBEC cytidine deaminases (signatures 2 and 13), and HRD (signature 3) predominated in tumor samples (Fig. 2) [31]. The contribution of signature 3 was significantly more pronounced in triple-negative breast cancer (TNBC) (Figure S2B) [32]. APOBEC deregulation was predominant in ERBB2-overexpressing cases (Figure S2C) [33]. The HRD score was higher in TNBC than in the other subgroups (Figure S2D). This score correlated with the relative contribution of signature 3 (*R*^2^ = 0.13, *p* = 0.002, Figure S2A). Significantly, CABR95, carrying the germline pathogenic *PALB2* mutation enriched by somatic LOH, had a high mean HRD score, in keeping with the expected effect of loss-of-function of this gene. MutSigCV identified significantly greater-than-expected somatic mutation rates of *TP53*, *PIK3CA*, and *GATA3* (Table S3) [34].

### Additional support for the pathogenic effect of somatically enriched variants

#### Evaluation of splice site alterations

Three tumor samples had somatic enrichment of a germline variant predicted to alter splicing. Using cDNA synthesized on RNA extracted from blood cells, we confirmed that the *MRE11A* germline variant enriched by LOH in the tumor of CABR61 altered normal splicing (NM_005591 c.1501-8 T>G, unknown in GnomAD). The resulting transcript retains eight intronic nucleotides upstream of exon 14, resulting in a frameshift (Figure S5). This tumor is furthermore associated with a high HRD score (Figure S2A). The *MUTYH* germline variant enriched by amplification in the tumor of CABR10 (c.1178G>A) is a hotspot pathogenic variant and has previously been shown to alter MUTYH function [35]. This tumor was however not characterized by an overaccumulation of G:C>T:A transversions (20%) or signature 18 contribution (reflecting 8-oxoguanine-related mutagenesis and MUTYH deficiency) [36]. The main contribution of signature 3 in this TNBC could not be explained by a studied genomic feature. RNA could not be obtained from CABR16 to test the *POLD3* germline variant, somatically enriched by amplification.

#### Immunohistochemistry to confirm local loss of expression

CABR74 carried a germline frameshift mutation in *PMS1* predicted to lead to nonsense-mediated mRNA decay. The matched tumor displayed CN-LOH significantly enriching the germline variant. Bi-allelic loss of PMS1 was confirmed by immunohistochemistry in the infiltrating tumor area of the sample, whereas normal tissue retained PMS1 expression (Figure S6).

#### Activating effect of a somatically enriched germline ERBB2 variant

**Fig. 3:**
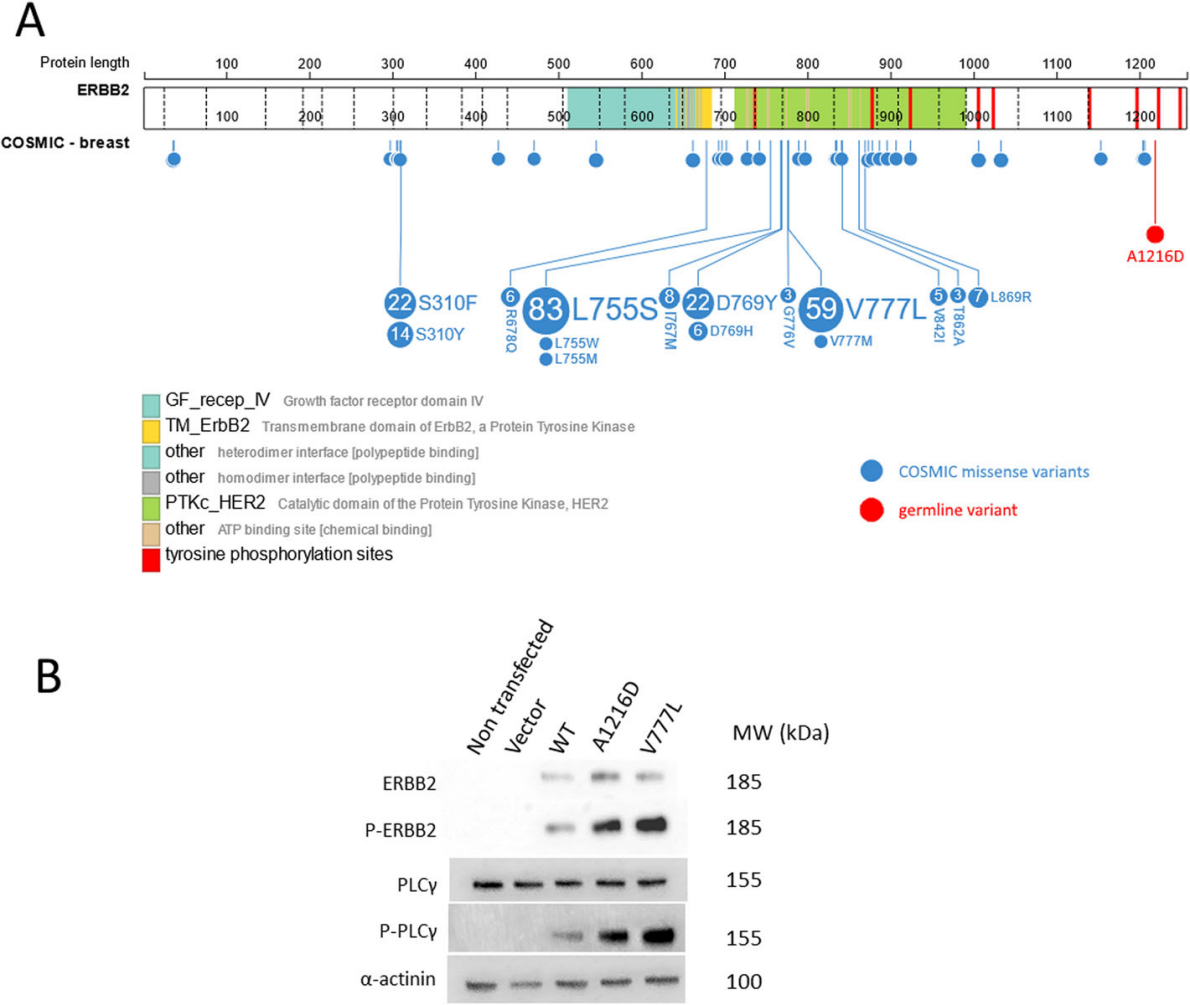
**a** Localization of the ERBB2 germline variant found in CABR74, as well as the somatic missense variants in COSMIC breast cancer samples. Figure adapted from ProteinPaint. **b** Western blot results of lysates of HEK293T cells transfected with designated ERBB2 constructs

CABR74 carried a germline *ERBB2* variant: c.3647C>A, encoding for p.A1216D. This variant was enriched to an almost homozygous state in the matched tumor, by CN-LOH. The change is present in GnomAD at an AF of 0.76%, with only one homozygous patient, predicted damaging by 3 algorithms and located in the C-terminal intracellular part of the protein, in the immediate neighborhood of established phosphorylation sites (Fig. 3a). Similar to the known activating mutation V777L, used as a positive control, we showed that the variant form of ERBB2, and the downstream signaling protein PLCγ, had strongly increased phosphorylation when transiently overexpressed in HEK293T cells (Fig. 3b).

#### Confirmation by WES performed in a second affected family member

Both germline and tumor DNA were obtained from an affected relative of CABR61 (with the germline *MRE11A* splice site mutation enriched in tumor) and CABR95 (with the germline *PALB2* pathogenic mutation inducing a frameshift and enriched in the tumor). Clinical characteristics of these patients are summarized in Table S4. Two of the four germline variants identified in CABR61 were shared by the relative, including the *MRE11A* variant, which was also enriched in the relative’s tumor, by LOH (Figure S7). She did not have any other germline variant of interest. Of the three germline variants identified in CABR95, only *PALB2* was shared by the affected niece. Her ERBB2-overexpressing tumor did not however present a somatic second hit affecting *PALB2*, nor have a high HRD score.

#### Confirmation by WES performed in a second primary tumor

A germline *ABCD4* variant was enriched by CN-LOH in the BC sample of CABR18. However, no second hit in this gene was detected in a later-developed ovarian cancer of this patient. Conversely, a germline *NF2* variant, enriched in ovarian cancer, was depleted in the BC. No second hits were identified corresponding to the germline variants of CABR4 in her bilateral BC tumors.

#### Germline variants identified in multiple unrelated patients

Of the 287 unique variants, 38 were identified in at least two unrelated patients. Of these, 7 presented a second hit, but none more than once. The germline *RAD51B* missense variant NM_133509 c.728A>G has been described in HBC [37] but is probably a rare polymorphism. Although present in five different patients, it was enriched only once by LOH and has a MAF of 1.1% in the European non-Finnish population in GnomAD.

#### Multidisciplinary review of the variants

**Fig. 4:**
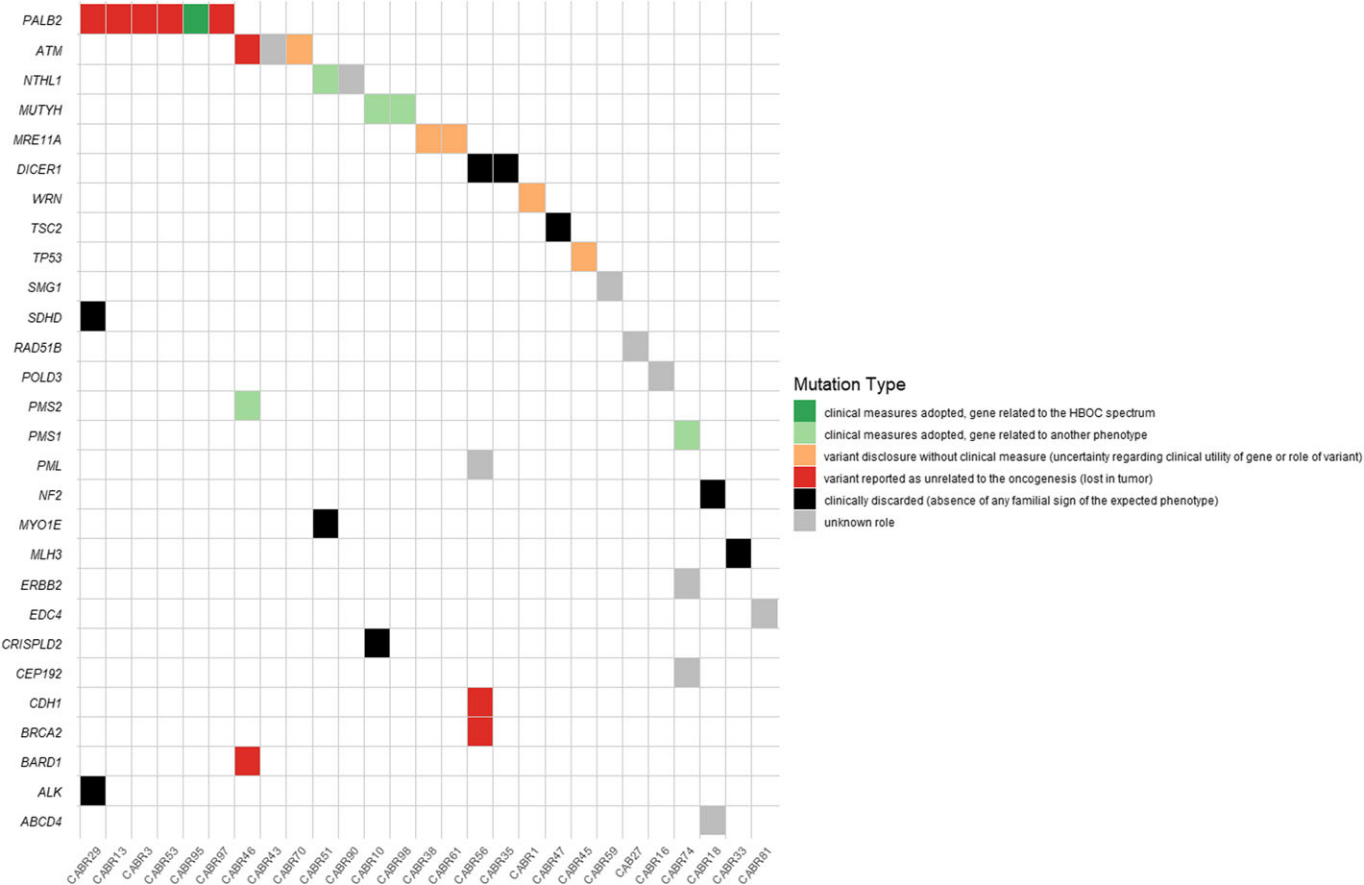
Conclusions adopted during the multidisciplinary discussion of the variants of interest. HBOC, hereditary breast or ovarian cancer

Each variant located in a gene with clinical involvement or presenting a second hit was reviewed by a multidisciplinary board of oncologists and geneticists to discuss whether the variant should be disclosed to the patient and could lead to clinical measures (Fig. 4 and Table S5). Of note, all our patients were already actively engaged in a BC screening program given their personal history. Thirty-nine variants found in 27 different patients were discussed, of which 12 variants in well-established BC-predisposing genes. The *PALB2* pathogenic variant led to clinical measures as recommended by the NCCN guidelines. In contrast, the *TP53* probably a pathogenic variant was conservatively disclosed without gene-specific clinical measures undertaken, as this family did not present the clinical spectrum of a penetrant *TP53* mutation and the second hit found is a frequent event in BC [38, 39]. Seven patients carried a variant on *ATM*, *BRCA2*, *CDH1*, or *PALB2* for which no argument pointed towards their implication in oncogenesis. We discussed 12 variants in cancer-predisposing genes unrelated or not related with certainty to HBOC. Clinical measures were cautiously discussed in five cases, taking mainly the familial features and their predisposing role to other cancer types into account, but not leading to HBOC risk prediction in their relatives. Several variants were discarded despite the presence of a second hit, as the clinical syndrome did not fit with the considered gene (*TSC2* (for which the somatic event is furthermore probably a passenger event), *DICER1*, *NF2*, *SDHD*, *ALK*, *CRISPLD2*, *MLH3*, *MYO1E*). In total, 11 patients (16% of the sequenced cohort) received personalized genetic information.

## Discussion

Multistage acquisition of DNA abnormalities in cancer-related genes is a well-recognized oncogenic process. Hereditary retinoblastoma and hereditary renal cell carcinoma (Von-Hippel Lindau disease) arise with the inheritance of a germline loss-of-function mutation in *Rb1* and *VHL*, respectively [40, 41]. The loss of the normal (functional) allele occurs locally, due to a somatic second mutation in the same gene, rendering these cells deficient. Inactivation of both *BRCA1* and *BRCA2* alleles appears to be required for the HRD characteristic of *BRCA*-related HBOC [22]. Two-hit inactivation has also been described, in smaller case series, for *PALB2*- and *ATM*-related HBC, and *BRIP1*-related hereditary ovarian cancer [42–44].

Our hypothesis was that matched tumor sequencing could be helpful in pinpointing genetic bases of suspected predisposition to BC in patients without pathogenic mutations in *BRCA1*, *BRCA2*, *TP53*, and *CHEK2*. In 735 cancer-related genes, we identified a mean of 4.7 variants per patient, with some in silico features of pathogenicity.

Of 329 germline variants, 28 from 19 different patients were significantly enriched in the paired tumor by a CNA, supporting a possible role for them in oncogenic processes. Importantly, CNA-related enrichment of these germline variants could not be attributed simply to an overall increase in genome-wide aneuploidy in these samples: cumulative aneuploidy size was not significantly different between samples that did or did not show a somatic second hit CNA at the locus of the germline variant. Besides, two genes presented a second somatic mutation, in samples not characterized by a high TMB. Twenty-five variants from 22 different patients were significantly depleted in the tumor sample by CNA, refuting the involvement of these variants in oncogenesis.

We showed that, besides gene-centric analyses, data on global somatic mutation patterns (TMB, somatic indel count, mutational signatures, and HRD) are necessary to refine the interpretation of germline variants. These analyses allow to differentiate somatic driver from passenger events and to highlight if the biological process related to the gene considered is dysregulated. These analyses confirmed the involvement and enrichment of the *NTHL1* variant in CABR51, similar to a previous study [45], whereas they helped to refute the role of another *NTHL1* variant in the oncogenesis of CABR90. In one case (CABR46), several arguments pointed towards the presence of a “WES-invisible” second hit mechanism involving the germline *PMS2* variant (e.g., gene promoter methylation) leading to a hypermutated tumor. While lacking definitive proof of mismatch repair deficiency, we could not find any other event that could explain the very high TMB associated with this tumor. A large study demonstrated that *BRCA1*, *BRCA2*, or *PALB2* (but not *ATM* or *CHEK2*) bi-allelic inactivation is associated with the mutational signature 3 [46]. In our study, high HRD scores could be explained in almost every case by tumor histology and molecular classification (invasive medullary carcinoma or TNBC) or by the presence of a tumor-enriched germline variant in a gene implicated in homologous recombination (*PALB2* in CABR95). Interestingly, CABR61 presented a tumor with suspected bi-allelic *MRE11A* inactivation and had a high signature 3 activity. The *MRE11A* variant was probably not implicated in the tumorigenesis of CABR38, as this tumor did not contain a sign of HRD (Figure S2A). This adds relevant data to the study of Polak et al., which did not contain a case of *MRE11A* inactivation [46].

Sequencing of the second primary tumor was also helpful in reclassifying variants. It served as an argument to weaken the case for ABCD4 and NF2 as oncogenic drivers of CABR18, given the discordant results found in her breast and ovarian tumors. This analysis should be considered with caution for several reasons; to our knowledge, data on the consistency of second hits in multiple tumors in a single patient carrying predisposing mutations are scarce. Furthermore, sporadic tumors may arise in patients with germline predisposing mutations [47]. Thus, both tumors will not necessarily present the same founder oncogenic events. Nevertheless, this analysis strengthened the hypothesis that the *MRE11A* variant in CABR61 is indeed pathogenic, as an enriching second hit was also detected in the tumor of her mother.

Predisposition to cancer has historically been linked to the transmission of a heterozygous defective tumor suppressor gene, giving rise to oncogenesis after the inactivation of the second allele. However, recent publications demonstrated the involvement of germline defects in oncogenes also responding to the two-hit mechanism. In a study of more than 10,000 cases from 33 cancer types, high tumor expression of a germline variant in an oncogene (*AR*, *MET*, *RET*, *CBL*, and *PTPN11*) was found in 33 patients [5]. Inherited susceptibility to lung cancer has also been demonstrated in rare families with a germline *EGFR* mutation, the majority of them harboring a somatic second hit [48–50]. Somatic activating mutations of *ERBB2* represent a well-described mechanism driving oncogenesis in several cancer types. These mutations typically cluster in the extracellular ligand-binding and intracellular kinase domains, but transmembrane and juxtamembrane domain mutations have also been identified [28, 51]. Here, we describe a patient with a germline *ERBB2* variant undergoing highly significant somatic enrichment by CN-LOH. Despite its unusual location in the C-terminal part of the protein, the expression of this variant strongly increased phosphorylation of ERBB2 and the downstream signaling protein PLCγ. Added to its low frequency in the general population (MAF 0.76%, with only one homozygous individual), this suggests the variant is a weak activating mutation requiring a second hit for oncogenesis.

We believe that the clinical spectrum of the phenotype is still a critical point to acknowledge when considering the predisposing role of a variant. Recently, several studies focusing on mutation prevalence questioned the ability of guidelines for cancer genetic testing to detect mutation carriers [52, 53]. Nevertheless, the penetrance of disease-causing mutations may vary according to the testing indications, family history pattern, and the presence of other risk factors; underscoring cautious decision-making is required when highlighting variants in a gene not fitting the classical clinical syndrome [54].

The limitations of our study are those that are typically encountered by geneticists and oncologists in the clinical setting: First, in most of the families, we were not able to obtain germline and tumor DNA from other affected relatives due to cancer-related death or from a second primary tumor. As demonstrated in four cases, this can be very effective in reinforcing or weakening the candidacy of the findings in the index patient. Second, we did not study all possible second hit mechanisms (e.g., epigenetic modification). While read-outs such as mutational signatures, TMB, and HRD analyses can be surrogate markers of defects in particular (classes of) proteins, they do not provide complete information on the ultimate genetic causes. Third, a large, collaborative dataset would increase the probability of encountering each germline variant at least twice. Consistency in the behavior of the variant across tumors could be seen as a strong argument for its implication in oncogenesis. Fourth, although we argue that it would be a missed opportunity to not consider somatic events and patterns for refinement of variant analysis, we also agree that this should not be considered as a stand-alone argument, irrespectively of the existing ACMG criteria. Pathogenic *BRCA1/2* variants do not present LOH in all pancreatic cancers [55]. Fifth, sample purity and sequencing depth are critical factors in determining the sensitivity of the detection of somatic events. Although all our samples had a tumor purity estimate > 30% (median 55%), we acknowledge that higher coverage would have been beneficial for more accurate LOH detection in the samples with lower tumor purity. Finally, as cancer is a multistage process evolving over time, predisposition due to a germline mutation implies the second hit is an early event. Multiregional tumor sequencing or single-cell sequencing would be useful in unraveling the evolutionary history of the disease, distinguishing drivers from passenger somatic mutations. Theoretical methods to infer the timing of events using single DNA samples exist, but are based on broad assumptions about tumor clonality and apply only to gain (mutation, amplification) and not to loss (deletion, LOH) of information [56].

## Conclusion

Our study shows that, based on the double-hit theory, matched tumor sequencing is a useful tool to refine the interpretation of variants in cancer-predisposing genes in high-risk BC patients. Several patients benefited from this approach, which furthered our understanding of the genetic drivers of their predisposition to BC and resulted in the implementation of clinical measures or, alternatively, provided reassurance regarding the absence of the role of the variant in cancer initiation. From a clinical point of view, these measures should still be adapted to the recognized clinical utility of the gene. Nevertheless, deeper insight into the biological consequence of the germline and/or somatic events remains required in many cases, as many genes do not have a validated read-out to estimate the effect of a genetic variant.

## Supplementary information

**Additional file 1: Table S1.** List of the 735 genes selected for germline variant analysis. Data regarding familial syndrome was obtained from the supplementary data of Zhang et al. [15] and updated by literature query. **Table S2.** List of germline variants retained after filtering. Information about their characteristics and their allele-specific read depths in the tumor. **Table S3.** Assessment by MutSigCV of the statistical significance of the clustering of somatic mutations in putative cancer genes. **Table S4.** Clinical characteristics of the affected relatives from which both germline as well as tumor DNA could be obtained. **Table S5.** Details regarding the multidisciplinary discussion of variants of interest.

**Additional file 2: Figure S1**. Chromosome-wide analysis of difference in allele balance between tumor and normal (DAB), using all heterozygous SNVs located on the chromosome carrying the germline variant suspected of DAB in the tumor. A. Example of a confirmed case of DAB, involving the q-arm of chromosome 2. B. Example of a denied case of DAB, as no SNV on chromosome 16 passes the threshold of significance. **Figure S2**. Scatterplot of the relative contribution of mutational signature 3 and the HRD mean score for each tumor. IMC : invasive medullary carcinoma. A. Relative contribution of mutational signature 3 by clinical tumor subgroup. B. Relative contribution of added mutational signatures 2 and 13 by clinical tumor subgroup. C.HRD mean score by clinical tumor subgroup. **Figure S3.** Number of indels for each tumor sample. **Figure S4.** MSIsensor score of the tumor samples. The positive control is an independent breast cancer case with proven microsatellite instability (Schröder et al, unpublished data). **Figure S5.** Evaluation of the splicing alteration of MRE11A due to the germline variant c.1501-8T>G in CABR61. A.CABR61 MRE11A cDNA compared to the normal MRE11A cDNA reference. B.CABR61 MRE11A cDNA compared to the MRE11A cDNA reference and inclusion of the 8 intronic nucleotides upfront of exon 14. C. Graphic representation of the splicing alteration of MRE11A in CABR61. **Figure S6.** Detection of PMS1 expression by IHC : Loss of PMS1 expression is observed in the infiltrating tumor cells of CABR74 (A and B), while PMS1 expression is still detected in normal adjacent cells (C). PMS1 expression is observed in infiltrating tumor cells (D and E) and in normal adjacent cells (F) in a control case. Magnification : A and D , 5 X; B,C, E and F, 40 x. **Figure S7.** Copy number analysis of the tumor sample of CABR61 (A) and her affected relative (B) demonstrating LOH of the *MRE11A* locus.

## Data Availability

The datasets supporting the conclusions of this article are included within the article (and its additional files).
