# Supplementary material for: Tumor sequencing is useful to refine the analysis of germline variants in unexplained high-risk breast cancer families
Source: Breast Cancer Res. 2020 Apr 15;22:36. doi: 10.1186/s13058-020-01273-y (PMC7161277; doi:10.1186/s13058-020-01273-y)

Supplementary Figure S1

Chromosome-wide analysis of difference in allele balance between tumor and normal (DAB), using all heterozygous SNVs located on the chromosome carrying the germline variant suspected of DAB in the tumor.

A. Example of a confirmed case of DAB, involving the q-arm of chromosome 2.

B. Example of a denied case of DAB, as no SNV on chromosome 16 passes the threshold of significance.

A

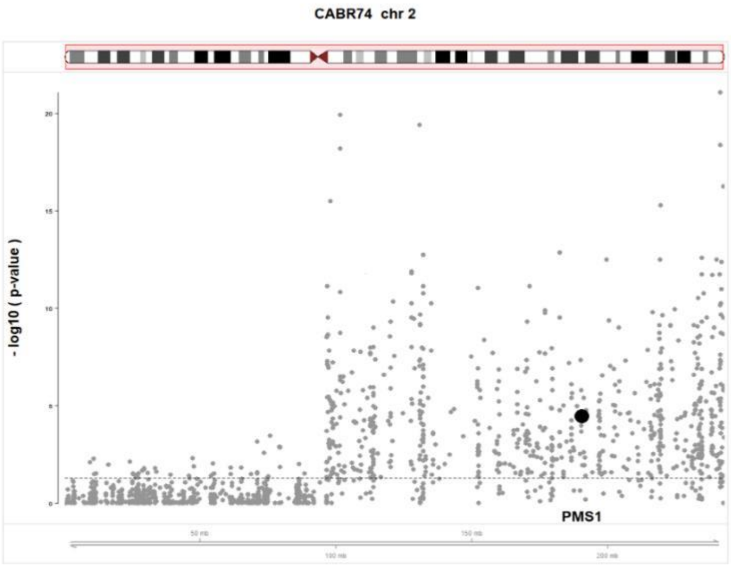

B

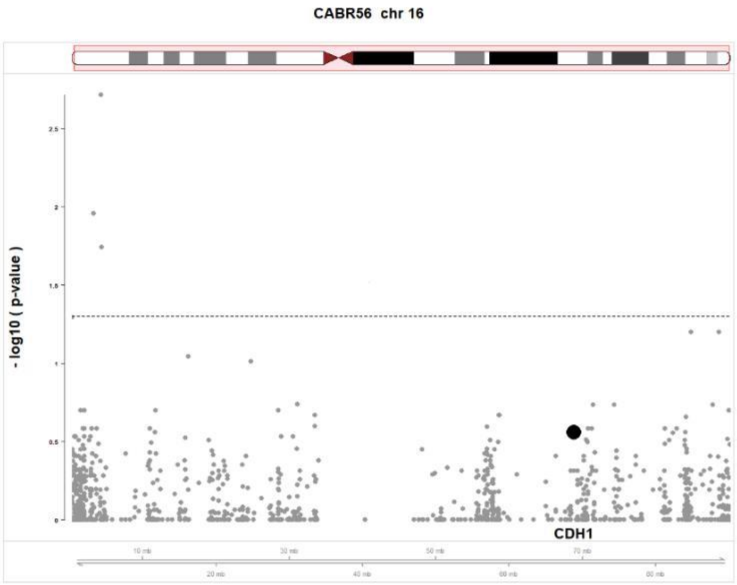

Supplementary Figure S2

A. Scatterplot of the relative contribution of mutational signature 3 and the HRD mean score for each tumor. IMC : invasive medullary carcinoma.

B. Relative contribution of mutational signature 3 by clinical tumor subgroup.

C. Relative contribution of added mutational signatures 2 and 13 by clinical tumor subgroup.

D. HRD mean score by clinical tumor subgroup.

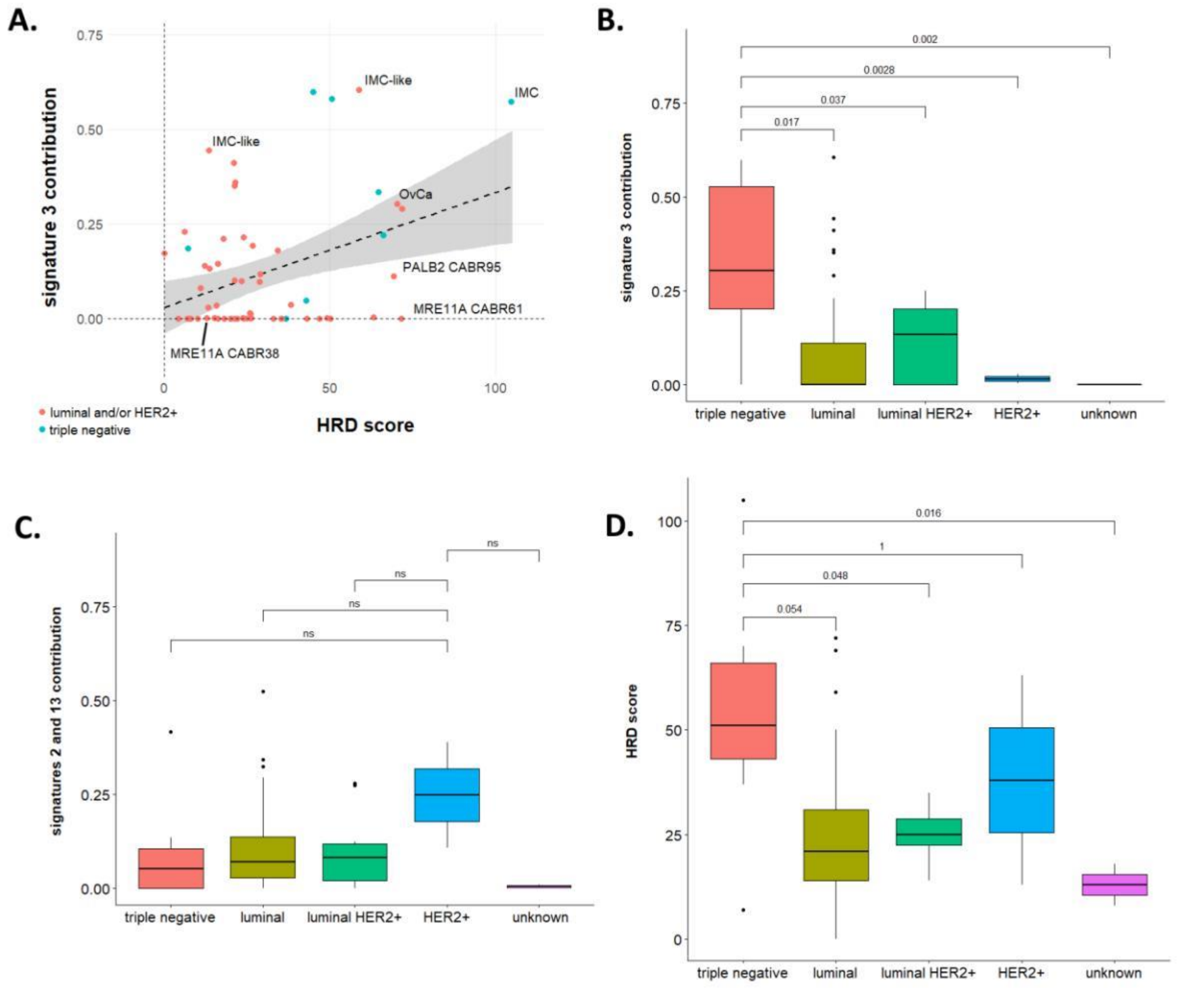

Supplementary figure S3

Number of indels for each tumor sample.

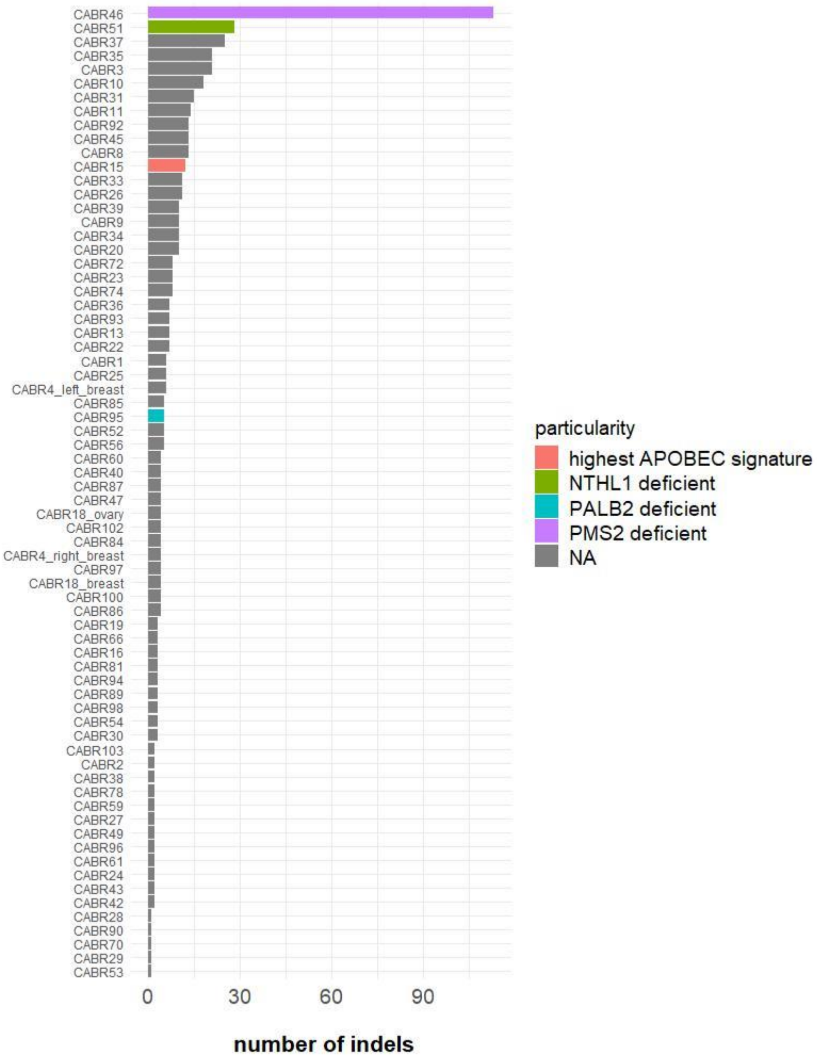

Supplementary figure S4

MSIsensor score of the tumor samples. The positive control is an independent breast cancer case with proven microsatellite instability (Schröder et al, unpublished data).

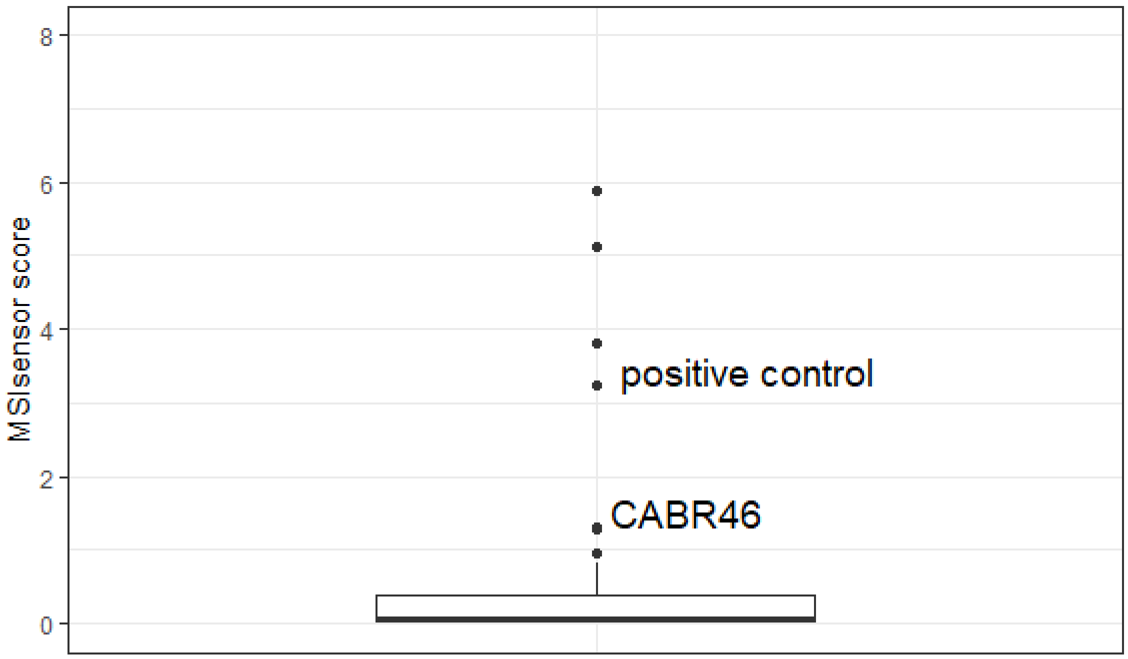

Supplementary figure S5

Evaluation of the splicing alteration of MRE11A due to the germline variant c.1501-8T>G in CABR61.

A. CABR61 MRE11A cDNA compared to the normal MRE11A cDNA reference.

B. CABR61 MRE11A cDNA compared to the MRE11A cDNA reference and inclusion of the 8 intronic nucleotides upfront of exon 14.

C. Graphic representation of the splicing alteration of MRE11A in CABR61.

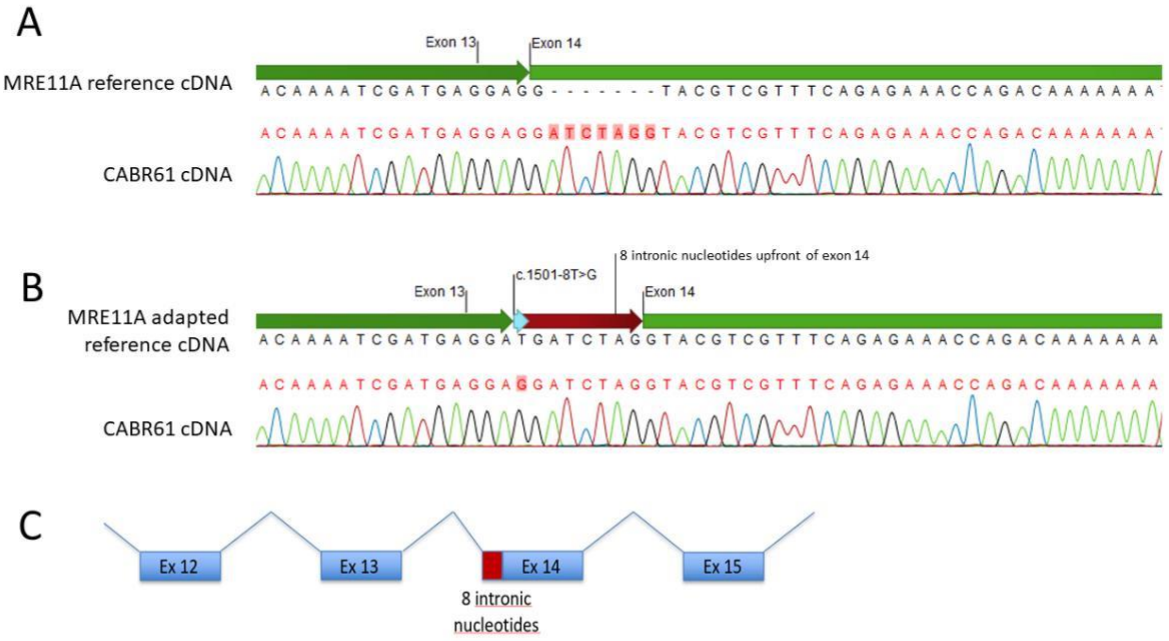

Supplementary figure S6

Detection of PMS1 expression by IHC : Loss of PMS1 expression is observed in the infiltrating tumor cells of CABR74 (A and B), while PMS1 expression is still detected in normal adjacent cells (C). PMS1 expression is observed in infiltrating tumor cells (D and E) and in normal adjacent cells (F) in a control case. Magnification : A and D , 5 X; B,C, E and F, 40 x.

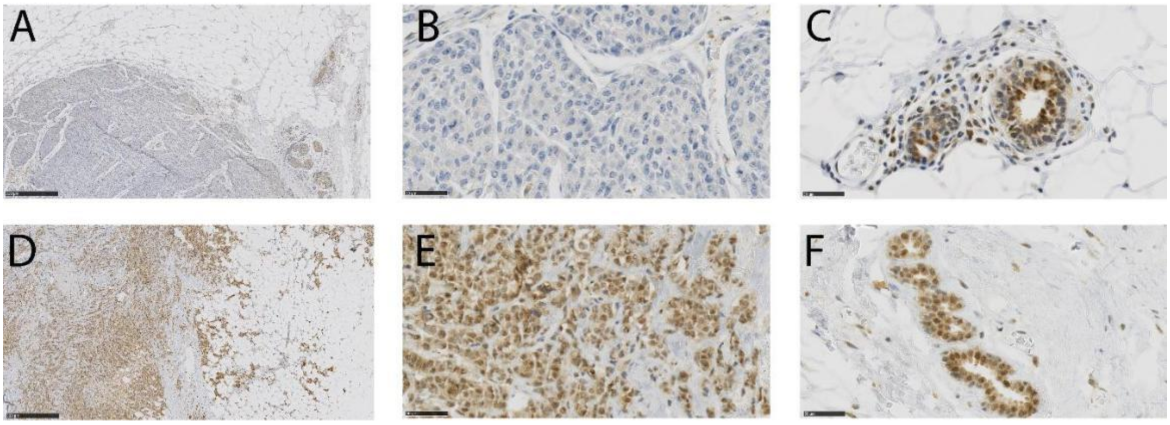

Supplementary Figure S7

Copy number analysis of the tumor sample of CABR61 (A) and her affected relative (B) demonstrating LOH of the *MRE11A* locus.

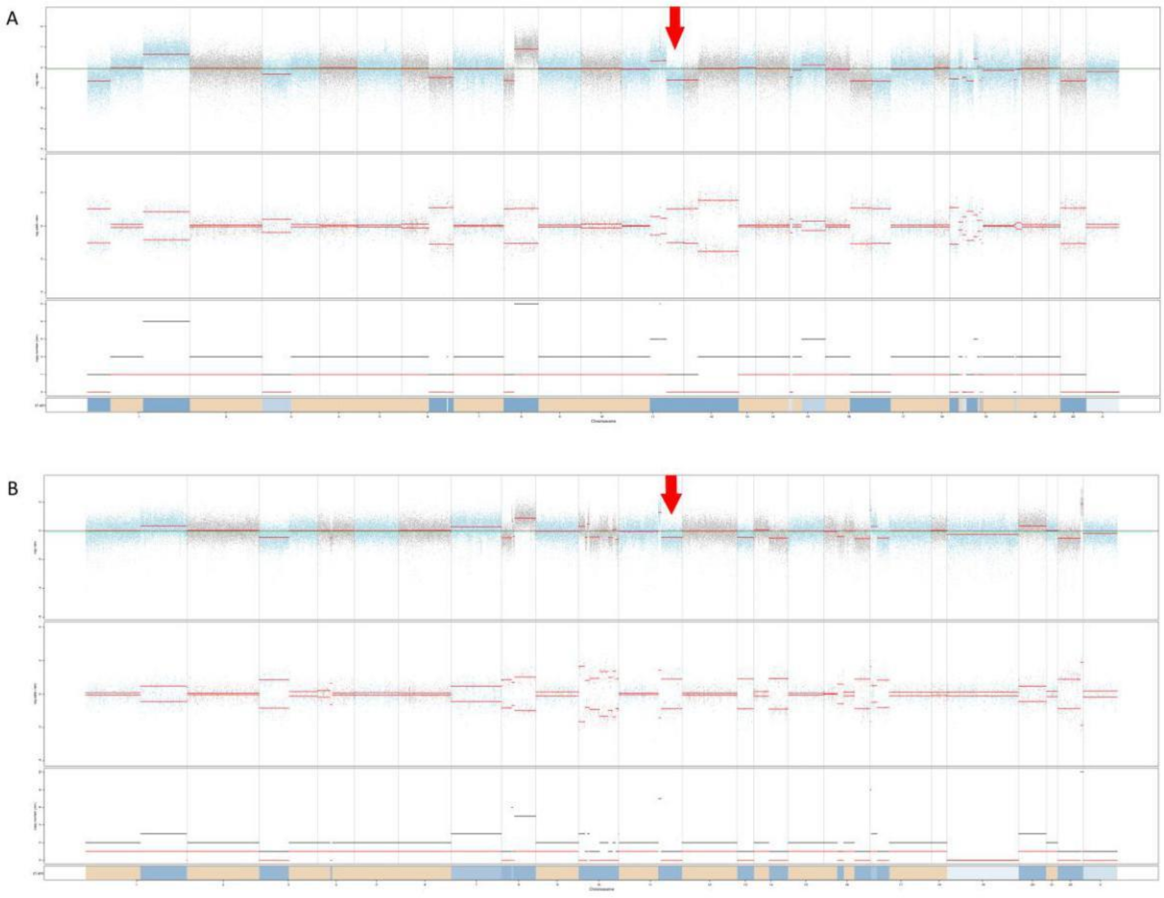

Supplement: Supplementary file 2 — Additional file 2: Figure S1. Chromosome-wide analysis of difference in allele balance between tumor and normal (DAB), using all heterozygous SNVs located on the chromosome carrying the germline variant suspected of DAB in the tumor. A. Example of a confirmed case of DAB, involving the q-arm of chromosome 2. B. Example of a denied case of DAB, as no SNV on chromosome 16 passes the threshold of significance. Figure S2. Scatterplot of the relative contribution of mutational signature 3 and the HRD mean score for each tumor. IMC : invasive medullary carcinoma. A. Relative contribution of mutational signature 3 by clinical tumor subgroup. B. Relative contribution of added mutational signatures 2 and 13 by clinical tumor subgroup. C.HRD mean score by clinical tumor subgroup. Figure S3. Number of indels for each tumor sample. Figure S4. MSIsensor score of the tumor samples. The positive control is an independent breast cancer case with proven microsatellite instability (Schröder et al, unpublished data). Figure S5. Evaluation of the splicing alteration of MRE11A due to the germline variant c.1501-8T>G in CABR61. A.CABR61 MRE11A cDNA compared to the normal MRE11A cDNA reference. B.CABR61 MRE11A cDNA compared to the MRE11A cDNA reference and inclusion of the 8 intronic nucleotides upfront of exon 14. C. Graphic representation of the splicing alteration of MRE11A in CABR61. Figure S6. Detection of PMS1 expression by IHC : Loss of PMS1 expression is observed in the infiltrating tumor cells of CABR74 (A and B), while PMS1 expression is still detected in normal adjacent cells (C). PMS1 expression is observed in infiltrating tumor cells (D and E) and in normal adjacent cells (F) in a control case. Magnification : A and D , 5 X; B,C, E and F, 40 x. Figure S7. Copy number analysis of the tumor sample of CABR61 (A) and her affected relative (B) demonstrating LOH of the MRE11A locus. [file 13058_2020_1273_MOESM2_ESM.pdf]
